# Past-Year Use Prevalence of Cannabidiol, Cannabigerol, Cannabinol, and Δ8-Tetrahydrocannabinol Among US Adults

**DOI:** 10.1001/jamanetworkopen.2023.47373

**Published:** 2023-12-13

**Authors:** Adrianne R. Wilson-Poe, Tristin Smith, Michael R. Elliott, Daniel J. Kruger, Kevin F. Boehnke

**Affiliations:** 1 Dow Neurobiology, Legacy Research Institute, Legacy Health, Portland, Oregon; 2 Chronic Pain and Fatigue Research Center, Anesthesiology Department, University of Michigan Medical School, Ann Arbor; 3 Biostatistics Department, University of Michigan School of Public Health, Ann Arbor; 4 Department of Community Health and Health Behavior, University at Buffalo, Buffalo, New York; 5 Jacobs School of Medicine and Biomedical Sciences, University at Buffalo, Buffalo, New York

## Abstract

This survey study characterizes past-year use prevalence and factors associated with use of cannabidiol, cannabigerol, cannabinol, and Δ8-tetrahydrocannabinol among US adults.

## Introduction

After passage of the 2018 Farm Bill, manufacturers have developed products derived from hemp (cannabis containing <0.3% Δ9-tetrahydrocannabinol [Δ9-THC]) featuring emerging cannabinoids, such as cannabidiol (CBD),^1^ Δ8-THC,^2^ cannabigerol (CBG),^3^ and cannabinol (CBN). It is currently unknown how widely these products are used. Our goal was to characterize past-year use prevalence and factors associated with use of CBD, Δ8-THC, CBG, and CBN among US adults.

## Methods

We conducted a cross-sectional survey study of adults 18 years or older using the National Opinion Research Center (NORC) AmeriSpeak panel between June 22 and 26, 2023. This probability-based panel has a recruitment rate of 34% and consists of approximately 54 000 people representing 96% of US households. Participants were recruited by NORC and freely consented to participate. This investigation was reviewed and approved by the NORC institutional review board and considered an exempt study (federal exemption 2) by the University of Michigan institutional review board. We followed the AAPOR reporting guideline.

We assessed past-year cannabis, CBD, Δ8-THC, CBG, and CBN use and whether participants had heard of emerging cannabinoids in the past year (eAppendix in Supplement 1). These cannabinoids were selected based on recently published clinical reports.^1,2,3^ Using logistic regression models, we investigated associations of demographic and cannabis use characteristics with any emerging cannabinoid use and Δ8-THC use. All *P* values were from 2-sided tests, and results were deemed statistically significant at *P* < .05. Analyses included sampling weights such that estimates are representative of the US adult population with respect to sex, age, educational level, race and ethnicity, and region. Analyses were performed in R, version 4.1.1 (R Project for Statistical Computing).

## Results

Overall, 1169 of 6666 panelists (17.5%) to whom the survey was released completed it (AAPOR research response rate, 2.9%). Among the participants (51.7% women; median age, 48 years [IQR, 33-63 years]), 26.3% reported past-year cannabis use (Table 1). Overall, 71.7% participants heard of CBD compared with 41.2%, 18.4%, and 16.8% for Δ8-THC, CBG, and CBN, respectively. Similarly, 21.1% reported past-year CBD use compared with 11.9%, 5.2%, and 4.4% for Δ8-THC, CBG, and CBN, respectively; 25.2% of participants reported past-year use of any emerging cannabinoid. Past-year cannabis use was associated with higher odds of using any emerging cannabinoid (odds ratio [OR], 23.9 [95% CI, 14.2-40.2]; *P* < .001), whereas non-Hispanic Black race (OR, 0.36 [95% CI, 0.15-0.88]; *P* = .02) was associated with lower odds of using any emerging cannabinoid (Table 2). Residing in states with medical (OR, 0.44 [95% CI, 0.21-0.90]; *P* = .02) or recreational (OR, 0.45 [95% CI, 0.22-0.91]; *P* = .03) cannabis laws was negatively associated with Δ8-THC use.

**Table 1.** Respondent Characteristics by Self-Reported Past-Year Cannabis Use (Unweighted N = 1142)^a^

Characteristic	Total	Past-year use of any emerging cannabinoid^b^	No past-year use of any emerging cannabinoid	Past-year use of Δ8-THC^c^	No past-year use of Δ8-THC
Sex (weighted %)					
Male	48.3 (44.2-52.4)	49.1 (40.6-57.6)	48.1 (43.5-52.7)	50.5 (37.4-63.6)	48.4 (44.1-52.7)
Female	51.7 (47.6-55.8)	50.9 (42.4-59.4)	51.9 (47.3-56.5)	49.5 (36.4-62.6)	51.6 (47.3-55.9)
Age, y					
Median (IQR)	48 (33-63)	40 (29-55)	50 (34-65)	34 (27-45)	50 (34-64)
Age (weighted %), y					
18-39	37.1 (33.0-41.2)	46.8 (38.2-55.4)	33.8 (29.1-38.5)	62.2 (49.4-75.0)	33.9 (29.6-38.2)
≥40	62.9 (58.8-67.0)	53.2 (44.6-61.8)	66.2 (61.5-70.9)	37.8 (25.0-50.6)	66.1 (61.8-70.4)
Race (weighted %)					
Hispanic	17.1 (13.7-20.5)	21.8 (13.8-29.8)	15.6 (12.0-19.2)	22.4 (10.0-35.0)	16.5 (13.1-19.9)
Non-Hispanic Black	11.9 (9.2-14.6)	10.6 (5.2-16.0)	12.3 (9.2-15.4)	14.4 (6.2-22.6)	11.6 (8.8-14.4)
Non-Hispanic White	61.8 (57.7-65.9)	60.9 (52.1-69.7)	62.0 (57.3-66.7)	53.0 (39.8-66.2)	62.7 (58.4-67.0)
Other^d^	9.2 (6.4-12.0)	6.6 (1.5-11.7)	10.1 (6.8-13.4)	10.2 (0.4-20.0)	9.1 (6.2-12.0)
Income (weighted %), $					
0-49 999	39.2 (35.2-43.2)	38.7 (30.5-46.9)	39.4 (34.8-44.0)	41.8 (29.6-54.0)	38.9 (34.7-43.1)
50 000-99 999	33.7 (29.8-37.6)	38.3 (29.6-47.0)	32.1 (27.8-36.4)	41.9 (28.2-55.6)	32.6 (28.6-36.6)
≥100 000	27.1 (23.6-30.6)	23.0 (16.3-29.7)	28.5 (24.4-32.6)	16.3 (7.4-25.2)	28.4 (24.6-32.2)
Educational attainment (weighted %)					
High school or less	37.8 (33.6-42.0)	38.5 (29.7-47.3)	37.6 (32.8-42.4)	38.7 (25.3-52.1)	37.4 (33.0-41.8)
Some college or Associate’s degree	26.2 (23.0-29.4)	31.1 (23.5-38.7)	24.6 (21.2-28.0)	36.6 (24.4-48.8)	25.0 (21.8-28.2)
Bachelor’s degree	20.5 (17.5-23.5)	20.7 (14.5-26.9)	20.5 (17.0-24.0)	15.0 (7.9-22.1)	21.4 (18.1-24.7)
Postgraduate or professional degree	15.4 (12.4-18.4)	9.7 (5.0-14.4)	17.3 (13.6-21.0)	9.7 (1.9-17.5)	16.2 (12.9-19.5)
Cannabis legal status (weighted %)^e^					
Recreational	28.1 (24.5-31.7)	30.1 (22.1-38.1)	27.4 (23.3-31.5)	41.5 (28.8-54.2)	26.6 (22.8-30.4)
Medical only	31.1 (27.3-34.9)	23.7 (16.5-30.9)	33.7 (29.2-38.2)	24.0 (13.6-34.4)	32.3 (28.2-36.4)
Illicit	40.8 (36.8-44.8)	46.2 (37.7-54.7)	38.9 (34.4-43.4)	34.5 (21.3-47.7)	41.4 (37.2-45.6)
Past-year cannabis use (weighted %)					
Yes	26.3 (22.6-30.0)	70.4 (62.4-78.4)	11.4 (8.3-14.5)	79.9 (67.5-92.3)	19.2 (15.7-22.7)
No	73.7 (70.0-77.4)	29.6 (21.6-37.6)	88.6 (85.5-91.7)	20.1 (7.7-32.5)	80.8 (77.3-84.3)

Abbreviation: Δ8-THC, Δ8-tetrahydrocannabinol.

^a^ Of the 1169 participants who completed the survey, 8 were excluded for skipping the question asking about past-year Δ8-THC use, and 19 were excluded for skipping or answering “don’t know” to the question asking about past-year cannabis use, leaving a preweighted analytical sample of 1142. Participant responses were weighted such that estimates are representative of included states.

^b^ Refers to use of any of the following compounds: cannabidiol, Δ8-THC, cannabigerol, or cannabinol.

^c^ Includes only participants who used Δ8-THC.

^d^ Other non-Hispanic, 2 or more races non-Hispanic, and Asian-Pacific Islander non-Hispanic.

^e^ Refers to the current legality of cannabis dispensaries (recreational or medical) in a participant’s state of residence.

**Table 2.** Associations of Demographic Characteristics With Past-Year Cannabis Use^a^

Characteristic	Past-year use of any emerging cannabinoid, OR (95% CI)^b^	*P* value	Past-year use of Δ8-THC, OR (95% CI)^c^	*P* value
Sex				
Male	1 [Reference]	.22	1 [Reference]	.24
Female	0.74 (0.45-1.20)		0.69 (0.36-1.29)	
Age, y				
18-39	1.26 (0.75-2.10)	.38	2.38 (1.18-4.84)	.02
≥40	1 [Reference]		1 [Reference]	
Race				
Hispanic	1.33 (0.59-2.98)	.50	1.48 (0.50-4.42)	.48
Non-Hispanic Black	0.36 (0.15-0.88)	.02	0.63 (0.23-1.73)	.37
Non-Hispanic White	1 [Reference]	NA	1 [Reference]	NA
Other^d^	0.57 (0.28-1.16)	.12	1.18 (0.52-2.66)	.70
Income, $				
0-49 999	1 [Reference]	NA	1 [Reference]	NA
50 000-99 999	1.41 (0.69-2.87)	.34	1.31 (0.52-3.33)	.57
≥100 000	1.27 (0.63-2.56)	.50	0.93 (0.43-2.00)	.85
Educational attainment				
High school or less	1.22 (0.53-2.84)	.64	0.77 (0.25-2.40)	.65
Some college or Associate’s degree	1.78 (0.80-3.93)	.16	1.24 (0.53-2.93)	.62
Bachelor’s degree	1.98 (0.90-4.38)	.09	0.80 (0.30-2.14)	.66
Postgraduate or professional degree	1 [Reference]	NA	1 [Reference]	NA
Cannabis legal status				
Recreational	0.93 (0.31-1.62)	.81	0.45 (0.22-0.91)	.03
Medical only	0.56 (0.54-1.02)	.06	0.44 (0.21-0.90)	.02
Illicit	1 [Reference]	NA	1 [Reference]	NA
Past-year cannabis use				
Yes	23.9 (14.2-40.2)	<.001	16.5 (6.6-40.9)	<.001
No	1 [Reference]	NA	1 [Reference]	NA

Abbreviations: Δ8-THC, Δ8-tetrahydrocannabinol; NA, not applicable.

^a^ Logistic regression models included all covariates in the same model.

^b^ Refers to use of any of the following compounds: cannabidiol, Δ8-THC, cannabigerol, and cannabinol.

^c^ Includes only participants who used Δ8-THC.

^d^ Other non-Hispanic, 2 or more races non-Hispanic, and Asian-Pacific Islander non-Hispanic.

## Discussion

We provide the first estimates, to our knowledge, of past-year use prevalence of CBN, Δ8-THC, and CBG in the US. A Gallup poll reported that 14% of US adults personally used CBD in 2019^1^; our reported 21% use prevalence of CBD represents a 50% increase over the past 4 years. Prevalence of past-year cannabis use was somewhat higher than in other studies but was similarly associated with younger age,^4^ and past-year cannabis use was also associated with using emerging cannabinoid products. Higher Δ8-THC use in states without medical or adult-use cannabis laws suggests that cannabis prohibition may unintentionally promote Δ8-THC use. There are few controlled human studies with emerging cannabinoids, but surveys suggest these products are used for treating sleep or pain^1^ and in place of other drugs, including pain medications.^2^

Based on these results, we support ongoing public health surveillance efforts targeting emerging cannabinoids because of lack of industry standards to protect consumers and similar pharmacology or effects of Δ9-THC and its hemp-derived impairing analogues (eg, Δ8-THC), which may be of particular concern for adolescents and young adults.^5^ Study limitations included not assessing emerging cannabinoid use patterns (eg, dose and use frequency) and possible sampling biases, although NORC implements probability-based recruitment best practices for their AmeriSpeak panel.^6^ Our results highlight the importance of future research to better understand perceptions of safety, motivations for use, and outcomes of use of these products.

## Notes

Supplement 1. **eAppendix.** Survey Questions

Supplement 2.
Data Sharing Statement
