# Supplementary material for: Past-Year Use Prevalence of Cannabidiol, Cannabigerol, Cannabinol, and Δ8-Tetrahydrocannabinol Among US Adults
Source: JAMA Netw Open. 2023 Dec 13;6(12):e2347373. doi: 10.1001/jamanetworkopen.2023.47373 (PMC10719758; doi:10.1001/jamanetworkopen.2023.47373)
Supplement: Supplement 1. — eAppendix. Survey Questions [file jamanetwopen-e2347373-s001.pdf]

Supplementary Online Content

Wilson-Poe AR, Smith T, Elliott MR, Kruger DJ, Boehnke KF. Past-year use prevalence of cannabidiol, cannabigerol, cannabinol, and Δ8-tetrahydrocannabinol among US adults. *JAMA Netw Open.* 2023;6(12):e2347373. doi:10.1001/jamanetworkopen.2023.47373

eAppendix. Survey Questions

This supplementary material has been provided by the authors to give readers additional information about their work.

© 2023 Wilson-Poe AR et al. *JAMA Network Open.*

eAppendix. Survey Questions

MI1.
Have you used cannabis (also known as marijuana) in the past year?

RESPONSE OPTIONS:
01 Yes
02 No

MI2.
For what reasons have you used cannabis in the past year?

RESPONSE OPTIONS:
01 Only for medical reasons
02 Only for non-medical reasons, such as relaxation, socially, or recreationally
03 For both medical and non-medical reasons

MI3.
In the past 30 days, how frequently have you used cannabis products of any kind (including smoking, eating, vaporizing, using topicals, or taking oils)?

RESPONSE OPTIONS:
01 Once
02 2 to 3 times
03 Once a week
04 2 to 3 days per week
05 4 to 6 days per week
06 Once or twice a day
07 Three or more times per day

MI4.
There are many compounds that come from cannabis. Have you heard of any of the following compounds?

A. Delta-8-THC
B. CBD
C. CBG
D. CBN

Response options for A-D
01 Yes
02 No

© 2023 Wilson-Poe AR et al. *JAMA Network Open.*

MI5.
In the past 12 months, have you used any of the following compounds from cannabis?

A. Delta-8-THC
B. CBD
C. CBG
D. CBN

Response options for A-D

01 Yes
02 No

© 2023 Wilson-Poe AR et al. *JAMA Network Open.*
