# Supplementary material for: Past-Year Use Prevalence of Cannabidiol, Cannabigerol, Cannabinol, and Δ8-Tetrahydrocannabinol Among US Adults
Source: JAMA Netw Open. 2023 Dec 13;6(12):e2347373. doi: 10.1001/jamanetworkopen.2023.47373 (PMC10719758; doi:10.1001/jamanetworkopen.2023.47373)
Supplement: Supplement 2. — Data Sharing Statement [file jamanetwopen-e2347373-s002.pdf]

Data Sharing Statement

Wilson-Poe. Past-Year Use Prevalence of Cannabidiol, Cannabigerol, Cannabinol, and Δ8-Tetrahydrocannabinol Among US Adults. *JAMA Netw Open*. Published December 13, 2023. doi:10.1001/jamanetworkopen.2023.47373

Data
Data available: Yes
Data types: Deidentified participant data
How to access data: Email kboehnke@med.umich.edu
When available: With publication

Supporting Documents
Document types: None

Additional Information
Who can access the data: Researchers whose proposed use of data has been approved
Types of analyses: Specified purpose
Mechanisms of data availability: With signed data access agreement
